# A Cross-Sectional Study Comparing Patient Information Guides Generated by ChatGPT and Google Gemini for Common Radiological Procedures

**DOI:** 10.7759/cureus.74876

**Published:** 2024-11-30

**Authors:** Vidith Phillips, Nidhi L Rao, Yashasvi H Sanghvi, Maryam Nizam

**Affiliations:** 1 Internal Medicine, Division of Biomedical Informatics and Data Science, Johns Hopkins University, School of Medicine, Baltimore, USA; 2 Internal Medicine, KAP Viswanatham Government Medical College, Tiruchirappalli, IND; 3 Internal Medicine, BJ Medical College, Ahmedabad, IND; 4 Emergency Medicine, Valaichchenai Base Hospital, Valaichchenai, LKA

**Keywords:** abdominal ct, abdominal ultrasound, artificial intelligence, chatgpt, educational tool, google gemini, mri of abdomen, patient education brochure

## Abstract

Introduction: Artificial intelligence (AI) plays a significant role in creating brochures on radiological procedures for patient education. Thus, this study aimed to evaluate the responses generated by ChatGPT (San Francisco, CA: OpenAI) and Google Gemini (Mountain View, CA: Google LLC) on abdominal ultrasound, abdominal CT scan, and abdominal MRI.

Methodology: A cross-sectional original research was conducted over one week in June 2024 to evaluate the quality of patient information brochures produced by ChatGPT 3.5 and Google Gemini 1.5 Pro. The study assessed variables including word count, sentence count, average words per sentence, average syllables per sentence, grade level, and ease score using the Flesch-Kincaid calculator. Similarity percentage was evaluated using Quillbot (Chicago, IL: Quillbot Inc.), and reliability was measured using the modified DISCERN score. Statistical analysis was conducted using R version 4.3.2 (Vienna, Austria: R Foundation for Statistical Computing).

Results: There is no significant difference between sentence count (p=0.8884), average words per sentence (p=0.1984), average syllables per sentence (p=0.3868), ease (p=0.1812), similarity percentage (p=0.8110), and reliability score (p=0.6495). However, there is a statistically significant difference, with ChatGPT having a higher word count (p=0.0409) and grade level (p=0.0482) than Google Gemini. P-values <0.05 were considered significant.

Conclusions: Both ChatGPT and Google Gemini demonstrate the ability to generate content that maintains consistency assessed through readability and reliability scores. Nevertheless, the noticeable disparities in word count and grade level underscore a crucial area for improvement in customizing content to accommodate varying levels of patient literacy.

## Introduction

Abdominal magnetic resonance imaging (MRI) is a noninvasive procedure that utilizes strong magnets and radio waves to create detailed images of the abdomen without using ionizing radiation (X-rays). In contrast, a computed tomography (CT) scan uses X-rays to generate cross-sectional images of the abdomen. An abdominal ultrasound employs sound waves to visualize internal structures within the abdomen.

Patient education plays a crucial role in healthcare, providing individuals with relevant information about their condition, symptoms, available treatments, expected outcomes, potential side effects, and preventive measures. This knowledge aids in early detection of complications, adherence to medications, and ultimately helps in preventing complications or reducing mortality and morbidity risks. This principle also applies to radiological procedures.

Using artificial intelligence (AI) for patient education offers distinct advantages. AI tools can analyze large datasets, detect patterns, and often outperform humans in various healthcare tasks. They enhance accuracy, reduce costs, save time, and ensure access to current information about radiological procedures from anywhere [1]. However, there are drawbacks. AI may sometimes use unverified or questionable data, which could result in patient misinformation. Over-reliance on AI-generated recommendations could strain doctor-patient relationships and, in some cases, result in user confusion or harm [2,3].

ChatGPT (San Francisco, CA: OpenAI) (a conversational AI model) and Google Gemini (Mountain View, CA: Google LLC) (a multimodal AI developed by DeepMind and Google Research) are employed to create patient brochures explaining radiological procedures such as MRI, CT, and ultrasound of the abdomen. These AI tools leverage diverse internet sources to deliver understandable information on procedure indications, techniques, prerequisites, and potential post-procedural complications [4,5]. As this takes form, the question arises as to how reliable or how understandable it is to patients with literacy rates. This study explores such variables in the use of two models of AI (ChatGPT and Google Gemini) in producing patient brochures on radiological abdominal procedures.

This study aimed to compare ChatGPT and Google Gemini-generated responses for writing patient education guides on abdominal ultrasound, abdominal CT, and abdominal MRI, focusing on readability and ease of understanding.

## Materials and methods

Study design and setting

A cross-sectional original research study was conducted over one week in June 2024. Since no human participants were involved in this study, ethics committee approval was not taken.

Study tools

Three common investigations in radiology were selected, namely abdominal ultrasound, abdominal CT, and abdominal MRI. Two AI tools were selected namely ChatGPT 3.5 and Google Gemini 1.5 Pro for generating brochures for patient education on June 15, 2024 [4,6].

Different prompts were used - “write a patient education guide for abdominal ultrasound,” “write a patient education guide for abdominal CT” and “write a patient education guide for MRI of abdomen.” The responses generated were collected in a Microsoft Word (Redmond, WA: Microsoft Corp.) document, and subsequently graded using various tools, including the Flesch-Kincaid calculator, which was used for calculating word count, sentence count, ease of understanding, and readability of the information generated [7]. The similarity was checked using the Quillbot (Chicago, IL: Quillbot Inc.) plagiarism tool, and the reliability of scientific text was assessed using a modified DISCERN score, which is a scale used to assess the accuracy of scientific text and health information. It uses five questions that can be scored 0 or 1 in relation to the text, where a score of 5 points indicates high reliability and a score of 0 points indicates poor reliability [8,9].

Data analysis

For statistical analysis, the data were exported to a Microsoft Excel (Redmond, WA: Microsoft Corp.) sheet. Statistical analysis was done using R version 4.3.2 (Vienna, Austria: R Foundation for Statistical Computing). The responses generated by ChatGPT and Google Gemini were compared using an unpaired t-test. P-value <0.05 was considered significant. The correlation between ease score and reliability score was compared using Pearson’s coefficient of correlation.

## Results

A patient education brochure was generated using ChatGPT and Google Gemini for abdominal ultrasound, abdominal CT, and abdominal MRI.

Table 1 shows the characteristics of responses generated by ChatGPT and Google Gemini. There was no significant difference in the sentence count (p=0.8884), average word per sentence (p=0.1984), average syllables per word (p=0.3868), ease score (p=0.1812), similarity percentage (p=0.8110), and reliability score (p=0.6495) between ChatGPT and Google Gemini. However, the word count (p=0.0409) and grade level (p=0.0482) were statistically significant for ChatGPT-generated responses as compared to Google Gemini.

**Table 1:** Comparison of metrics between ChatGPT and Google Gemini. *A t-test was performed to obtain p-values. **P<0.05 was considered statistically significant.

Variables	ChatGPT		Google Gemini		p-Value*
	Mean	Standard deviation	Mean	Standard deviation	
Words	534.30	13.58	420.70	45.83	0.0409**
Sentences	41.00	7.81	40.00	8.54	0.8884
Average words per sentence	13.33	2.35	10.73	1.63	0.1984
Average syllables per word	1.77	0.06	1.70	0.10	0.3868
Grade level	10.43	0.65	8.67	0.84	0.0482**
Ease score	43.83	3.70	52.10	7.34	0.1812
Similarity percentage	51.27	28.44	46.60	11.27	0.8110
Reliability score	3.00	1.00	3.33	0.58	0.6495

**Figure 1:**
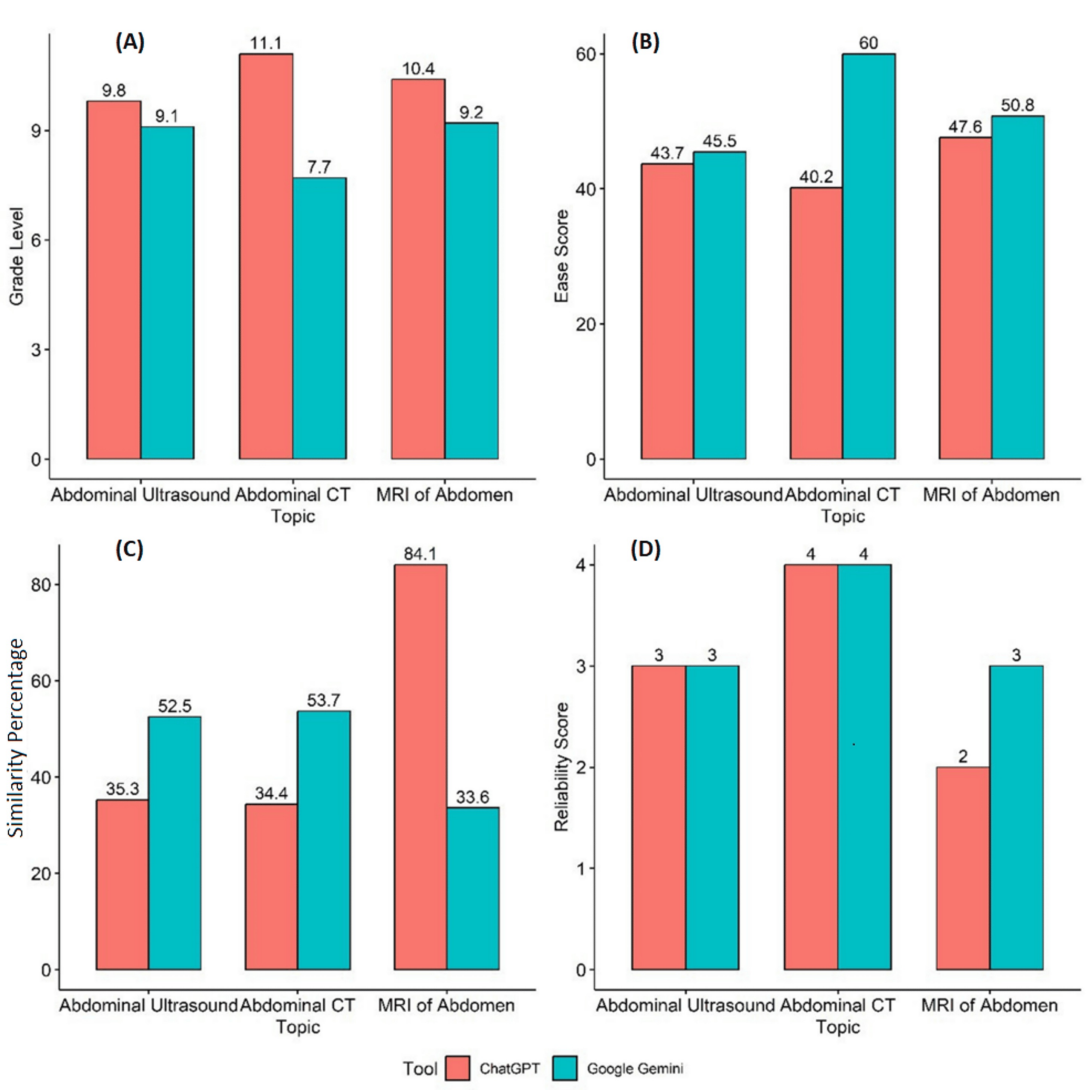
Graphical representation of comparison between grade level (A), ease score (B), similarity percentage (C), and reliability score (D) for the patient education guide generated by ChatGPT and Google Gemini.

Figures 1A-1D depict the graphical representation of the various scales used to assess the chatbots and the comparison of scores between ChatGPT and Google Gemini for each of the topics.

Figure 1A shows a graphical representation of the comparison between grade levels for the AI tools. ChatGPT scored higher than Google Gemini for all three topics, i.e., abdominal ultrasound (9.8), abdominal CT (11.1), and MRI of abdomen (10.4). The grade levels were found to be significantly associated (p=0.0482).

Figure 1B shows the ease score of the AI tools. ChatGPT scored lower for all three topics than Google Gemini, which scored 45.5 for abdominal ultrasound, 60 for abdominal CT, and 50.8 for MRI of abdomen. No significant difference between the AI tools was observed (p=0.1812).

Figure 1C shows similarity percentages. ChatGPT had a higher similarity percentage for MRI of abdomen (84.1%), while Google Gemini had a higher similarity percentage for abdominal ultrasound (52.5%), and abdominal CT (53.7%). No significant difference was observed (p=0.8110) between the mean scores.

Figure 1D shows reliability score. ChatGPT and Google Gemini scored equally for the topics of abdominal ultrasound and abdominal CT, while Google Gemini scored higher than ChatGPT for the topic of MRI of abdomen. No significant difference between the mean reliability scores was observed (p=0.6495).

## Discussion

The application of AI in patient education, as demonstrated in this study comparing ChatGPT and Google Gemini's capabilities, represents a significant advancement in healthcare communication.

This study’s findings indicate that AI, specifically ChatGPT, has proven effective in delivering high-quality, nuanced communications. This is consistent with findings by Ayers et al., who noted that AI could surpass traditional methods in providing empathetic responses to patient inquiries. This suggests that AI's potential to enhance patient satisfaction and adherence by addressing patients' emotional and informational needs is significant [10]. In this study, a significant difference in the readability levels between ChatGPT and Google Gemini has been noted, with ChatGPT generally producing content at a higher grade level. This echoes the findings of Rouhi et al., who demonstrated AI's ability to adjust medical content to appropriate literacy levels, thus making complex medical information more accessible and understandable [11].

Similar to the findings of Nazir et al., which suggest that different AI models are better suited for specific medical specialties, this study also highlights the importance of selecting the right AI tool to meet the unique educational needs of different patient groups. This selection is crucial for optimizing the effectiveness of patient education materials generated by AI [12]. Similar to how Ocakoglu and Coskun discussed AI's capability to produce detailed and accurate educational content for pelvic organ prolapse, this study assessed the comprehensiveness and precision of AI-generated materials for abdominal radiology. Both studies suggest that AI can effectively tailor information to enhance patient understanding [13]. Reflecting on the findings in the Mayo Clinic Proceedings (2024), this research supports the notion that AI can overcome language barriers and cultural differences, enhancing the global reach and effectiveness of patient education. This adaptability is particularly beneficial in diverse healthcare environments where patient populations have varied educational and cultural backgrounds [14]. Echoing findings by Chakravorti, this study also brings to light the ethical considerations necessary in the deployment of AI technologies. The need for transparency in how AI models are trained and how they handle patient data is critical for maintaining trust and ensuring the ethical use of AI in patient education [15].

The insights from this study, alongside corroborating evidence from recent research, underscore the need for ongoing evaluation and adaptation of AI tools in healthcare [16,17]. Future research should aim to address the identified gaps, such as the variation in readability and the need for more personalized content, to fully leverage AI's potential in patient education. Furthermore, there are several ethical and societal considerations surrounding AI applications in healthcare that warrant careful attention. Issues such as potential biases in AI algorithms, patient privacy concerns, and the need for transparency in AI-driven decisions are critical for ensuring equitable and trustworthy adoption. Moreover, the role of AI in complementing, rather than replacing, clinical judgment remains a significant area for ethical reflection. Future frameworks must address these challenges to align AI innovations with patient-centered care. Recent studies, including Gupta et al. and Ong et al., underscore ethical and regulatory challenges associated with AI, particularly large language models, in clinical diagnostics and medicine more broadly, emphasizing the need for robust ethical guidelines and regulatory oversight [18,19].

Limitations

Despite its insights, this study has several limitations that should be considered. First, the analysis was confined to a single-week period, which may not sufficiently capture the long-term consistency and variability of AI-generated content. A more extended study period could provide deeper insights into the reliability and effectiveness of AI tools over time. The study only included two AI tools, which may not represent the entire spectrum of AI capabilities currently available. Including a broader range of AI technologies might reveal different strengths and weaknesses, providing a more comprehensive understanding of the landscape of AI-generated patient education materials.

Furthermore, the study's focus was limited to patient education materials for abdominal radiological procedures, which might not be generalizable to other medical domains. Different types of medical procedures may require distinct approaches to patient education, influenced by specific medical terminologies and concepts that were not addressed in this study. The reliance on automated readability and reliability assessments, while valuable, does not capture the nuanced understanding and personal reactions of patients to the education materials. Future studies should include qualitative measures, such as patient feedback and expert evaluations, to assess the practical impact and effectiveness of AI-generated content in real-world settings.

Addressing these limitations in future research will be vital for advancing further understanding of the role of AI in healthcare communication and for optimizing the design and deployment of AI tools to meet the educational needs of patients effectively.

## Conclusions

This study provides a critical evaluation of the efficacy of AI tools, specifically ChatGPT and Google Gemini, in generating patient education materials for abdominal radiological procedures. This study reveals that both AI tools can produce materials that are consistent in quality, as measured by readability and reliability scores. However, the significant differences in word count and grade level between the tools highlight an important area for improvement - the adaptation of content to meet diverse patient literacy levels. This study underscores the potential of AI to enhance patient education by offering accurate, accessible, and timely information, which is crucial for informed patient decision-making and improved healthcare outcomes.

As AI technology continues to evolve, it presents a promising avenue for addressing the challenges of patient education across various medical fields. By leveraging AI, healthcare providers can potentially streamline the creation of educational content, ensuring it is both comprehensive and understandable. Continued research and development in this area are essential to fully realize the potential of AI in enhancing patient education and, consequently, patient engagement and satisfaction.
